# Dislocations of Femur Reduced by Manipulation, in London Hospitals

**Published:** 1856-03

**Authors:** 


### ECLECTIC DEPARTMENT,

### AND SPIRIT OF THE MEDICAL PERIODICAL PRESS.

---

*Dislocations of Femur Reduced by Manipulation, in London Hospitals.*— The London medical periodicals have recently contained reports of cases of reduction of dislocations of the femur, by manipulation. The first cases, two in number, occurred in Guy's Hospital, (Lancet, July 7,) under the care of Mr. Cock; one was of five week's standing, the other more recent; both had been subjected to the pullies. The reduction was easily effected in both cases by manipulation. The reporter adds, "from a careful study of the skeleton, Mr. Cock finds the secret to be, to handle the femur as if it were the humerus; then flexing it on the pelvis, abduct strongly, thus lifting the head of the bone with the accessory band of the ilio-femoral ligament, over the acetabulum, while you also rotate the lower condyle or limb, in your hand outward."

In the Lancet for Aug. 4, we find another report from Guy's Hospital, of seven cases under the care of Mr. Birkett, four of which were reduced by manual extension, and three by flexing the thigh upon the pelvis, abducting and rotating it. Of the three latter, two were in the foramen ovale, and one in the ischiatic notch. The writer makes the following introductory remarks:

"Dr. Reid, of New York, also gives an American plan of reduction in cases of dislocation of the head of the thigh-bone; and Dr. Markoe, one of the attending surgeons of the chief N. Y. Hospital, has recently furnished nine cases of his own, and three others treated by New York surgeons, twelve cases in all; of which three were dislocations into the foramen ovale, eight (five of his own and three of others,) on the dorsum ilii, and one into the ischiatic notch. We do not hear of the luxation on the horizontal branch of the os pubis, or the still more rare accident behind the tuberosity of the ischium. The method of the American surgeons is much more violent, and not more effective, than that adopted at Guy's and at some other London hospitals; the displacement being remedied, according to the former, 'by flexing the leg on the thigh, carrying the thigh over the sound one, upward over the pelvis, as high as the umbilicus, and then abducting and rotating it.' A peculiar 'rocking motion' is much dwelt on, however, by our transatlantic brethren, as necessary to induce the head of the bone to regain or creep back to its normal position,—a plan spoken of by Colombat—an old modification of the present methods by manipulation, also now highly prized by some surgeons; but we doubt if, under chloroform, the patient could be so well adapted to it as in the plan of Mr. Cock, and Mr. Birkett, at Guy's, which seems to answer all purposes."

If the writer of this report will carefully examine the valuable paper of

Dr. Reid, in the July, 1855, number of the *New York Jour. of Med.*, he will learn that the "American plan," or the method of Dr. Reid, is *entirely destitute* of violence. Dr. R. even deprecates the employment of chloroform, as "the pain produced by manipulation, is too trifling to require an anæsthetic of any kind." His third proposition, if followed, effectually prevents all violence: "The general rule for reducing dislocations should be, that that the limb or bone, should be carried, flexed, or drawn, in that direction which will relax the extended muscles."

Dr. Markoe's experience confirms this view; he says, "we never have accomplished anything by proceeding in a direction where great force was required to continue the movement, but have always succeeded by finding a direction in which the mere continuance of the movement without force, has brought the head into the proper position."

In the "American plan," again, the "rocking motion" has no existence. Dr. Reid speaks of "making gentle oscillations of the thigh," and condemns the rocking motion mentioned by Dr. Markoe.

In the present number of this journal, we supply the deficiency in the history of this operation, alluded to by our London contemporary. The two cases of dislocation upon the pubes, reported in our original department, by Dr. E. J. Fountain, of Iowa, are extremely interesting, and their successful treatment by manipulation alone, reflects great credit upon this ingenious and accomplished surgeon. The warmth of Dr. Fountain's praise of this great improvement in practical surgery, and his acknowledgment of Dr. Reid's merit are appropriate. We can well imagine the degree of estimation which the isolated practitioner must place upon a method of treatment of one of the most formidable accidents in surgery, which allows him to dispense with pullies, cords, bandages, anæsthetics, a half-score of assistants, and enables him, with facile hand to accomplish in twenty seconds, without pain, what before, with all these trappings, required hours, and even then often failed.

We deem it our duty to make this allusion to the negligence of foreign writers to accord to American surgery its proper due. It matters nothing how many times luxations of the femur have been accidentally or deliberately replaced by manipulation, to Dr. Reid belongs the unqualified merit of *first* establishing, by actual demonstration of its principles, the proper method of procedure. However much Mr. Cock, of Guy's Hospital, may have studied the skeleton for "the secret," he has not discovered a plan of reducing dislocations of the femur superior to the Americans. The first intimation of this method of operating, though not acknowledged, we doubt not was derived by the London surgeons, from the paper of Dr. Markoe, in the *New York Jour. of Med.*, for January, 1855, which was soon after republished in England.—*N. Y. Jour. of Med.*

